# Comparative Clinical Study Evaluating the Efficacy and Safety of Topical 5% Cetosomal Minoxidil and Topical 5% Alcohol-Based Minoxidil Solutions for the Treatment of Androgenetic Alopecia in Indian Men

**DOI:** 10.7759/cureus.46568

**Published:** 2023-10-06

**Authors:** Sandeep Sattur, Abhay Talathi, Geetanjali Shetty, Shehnaz Arsiwala, Rickson Pereira, Dhiraj Dhoot

**Affiliations:** 1 Department of Hair Restoration, Hairrevive – Center for Hair Restoration and Skin Rejuvenation, Mumbai, IND; 2 Department of Hair Restoration Surgery, Wockhardt Hospital, Mumbai, IND; 3 Dermatology, SkinSpace Clinic, Mumbai, IND; 4 Dermatology, Revitalis Clinic, Mumbai, IND; 5 Dermatology, Renewderm Skin Hair Laser Aesthetics Centre, Mumbai, IND; 6 Dermatology, Dr. Rickson's Dermatherapie Clinic, Mumbai, IND; 7 Dermatology, Holy Family Hospital, Mumbai, IND; 8 Global Medical Affairs, Glenmark Pharmaceuticals Ltd., Mumbai, IND

**Keywords:** india, hairdex-29, safety, efficacy, conventional minoxidil, cetosomal minoxidil, androgenetic alopecia

## Abstract

Introduction

Patients with androgenetic alopecia (AGA), who use alcohol-based topical minoxidil solutions, frequently experience localized irritation, dryness, and scalp redness. In this study, we compared the safety and effectiveness of topical 5% cetosomal minoxidil solution to those of topical 5% alcohol-based minoxidil solution in Indian men with AGA.

Methods

In this randomized, open-label study, male patients with AGA were randomized 1:1 to receive either solutions twice daily for 16 weeks. Efficacy endpoints included changes in basic and specific (BASP) grading, improvement in the trichoscopy score, and global photography at week 16 from baseline, whereas safety was evaluated by adverse events reported by patients and hair-related quality of life (QoL) using the Hairdex-29 questionnaire.

Results

Of the 80 patients, only 40 completed the study and were considered for complete analysis. Twelve out of 23 patients (52%) in the cetosomal minoxidil group and four out of 17 patients (24%) in the alcohol-based minoxidil group showed a positive increase in hair growth according to the trichoscopy score (p=0.1). According to the BASP grading system, nine patients (39%) and five patients (29%) in the cetosomal and alcohol-based minoxidil groups, respectively, showed improvement (p=0.73). Similarly, 19 (83%) and 10 (59%) patients in the cetosomal and alcohol-based minoxidil groups, respectively, reported positive hair growth on the global photography assessment (p=0.15). All the patients tolerated the treatment well, with no discontinuation in either group. There were four adverse events in the cetosomal minoxidil group, reported by two (9%) patients, whereas in the alcohol-based minoxidil group, 10 adverse events were reported by seven (41%) patients (p=0.02). In addition, the mean Hairdex-29 score of 40.26±4.71 at baseline improved to 32.32±3.35 in the cetosomal group, whereas it improved to 34.64±3.41 from 39.64±4.98 in the other group (p=0.03).

Conclusions

The 5% cetosomal minoxidil group showed improved safety but similar efficacy when administered twice daily. Therefore, cetosomal minoxidil may be a better option for treating AGA in males who are sensitive or nontolerant to alcoholic formulations.

## Introduction

Androgenetic alopecia (AGA) is the most common type of progressive hair loss disorder, which affects a great number of people globally, with the incidence increasing with age [1]. The most common age affected by AGA is around 30-50 years, with an increasing prevalence of approximately 58% in young men. This raises serious concerns because it not only has an impact on psychological health but also results in social anxiety disorders and depression [2].

The US Food and Drug Administration (FDA) has only approved minoxidil as a topical medication for hair regrowth [3]. It is a widely used over-the-counter product that is permitted for use in both men and women. However, the efficacy of minoxidil is poor. After 16 weeks of administration, minoxidil has been found to promote hair growth in approximately 35% of individuals in clinical trials [3,4].

While 30-40% of patients in physician-guided studies reported success, this percentage is dramatically lower in subjects who self-administer minoxidil. Compliance with the 16-week topical regimen remains a key obstacle to success [5]. Only 4% of 8,000 minoxidil users who participated in a consumer poll were happy with treatment outcomes [6]. Increasing the proportion of patients that respond to minoxidil could have a significant impact on both compliance and clinical benefit, given the amount of time needed to detect efficacy.

This might be because the bulk of the minoxidil solutions used propylene glycol, water, and alcohol as the carriers. Due to the inclusion of ethanol in their formulation, the majority of commercially available minoxidil treatments experience issues with restricted absorption, scalp irritation, dryness, hair frizzing, itching, and inflammation [7]. Recent studies have shown that non-alcohol-based minoxidil solutions have a higher safety profile than currently available formulations [2,8]. Recently, cetosome-based non-alcoholic minoxidil preparations have been commercialized in India as 5% minoxidil.

Currently, there is a scarcity of comparative clinical data on cetosome-based and alcohol-based minoxidil solutions (traditional minoxidil). Hence, this prospective clinical study aimed to assess the clinical efficacy and safety of both formulations for AGA management.

## Materials and methods

Eighty adult male patients with AGA of mild-to-moderate severity according to the basic and specific (BASP) grading for male AGA were enrolled in this comparative clinical study from five centers in Mumbai. Patients with other causes of alopecia, infections, autoimmune disorders, or other disorders affecting the scalp were excluded. All patients provided informed consent before enrollment in the study. This study was conducted from October 2021 to October 2022 and approved by an independent ethics committee (Suraksha Ethics Committee, regestration no. ECR/644/Inst/MH/2014/RR-20). This clinical study was performed in accordance with the Good Clinical Practice guidelines and the Declaration of Helsinki 1996 and was registered with the Clinical Trials Registry of India (CTRI/2021/09/036777). 

The patients were divided into two groups using a computerized randomization method. Group I received a novel 5% cetosomal minoxidil solution, and Group II received a 5% alcohol-based minoxidil solution. Both drugs were supplied by Glenmark Pharmaceuticals Ltd., India. All patients were instructed to apply the medicine twice a day for 16 weeks and refrain from using any other hair growth treatments. After the baseline visit, all the patients were advised to visit the investigator for follow-up at weeks 4, 8, and 16. At each follow-up visit, efficacy was measured by changes in the BASP grading, improvement in the trichoscopy score [9], and global photography assessment. Safety was evaluated based on adverse events reported by the patients at every visit, and hair-related quality of life (QoL) was recorded using the Hairdex-29 questionnaire [10] at baseline and at the end of treatment.

A circular area on the anterior leading edge of the vertex-balding scalp was chosen as the target area for trichoscopy, which was performed at baseline and weeks 8 and 16. The trichoscopy score was adapted from Pinto et al., as reported previously [9]. This score consisted of a rating of eight AGA symptoms, with higher scores indicating better outcomes (Table 1).

**Table 1:** Trichoscopy scoring system Adapted from Pinto et al. [9]

Sign		1 point	0 point
1	Number of hairs	More than a few	A few
2	Distance between follicles	Short	Mid to long
3	Miniaturized follicles	A lot	A few
4	Hair diameter	Diverse	All the same
5	Miniaturized and non-miniaturized hairs in the same follicular unit	Yes	No
6	Yellowish pigment	No	Yes
7	Inflammation	No	Yes
8	Scalp general condition	Good	Not good

Global photography was used to document hair loss in each patient at baseline and at weeks 8 and 16. At the completion of the study, global photographs were assessed by three independent reviewers with AGA expertise. Haidex-29, comprising 29 questions, captures hair-related QoL based on three subscales: emotion, function, and symptom (score range, 0-145) [10].

Statistical analyses were performed using IBM SPSS Statistics for Windows, version 20 (released 2011; IBM Corp., Armonk, New York, United States). The results are presented as mean scores and were compared using Student’s t-test and Fisher’s exact test with a level of significance of 0.05.

## Results

**Figure 1:**
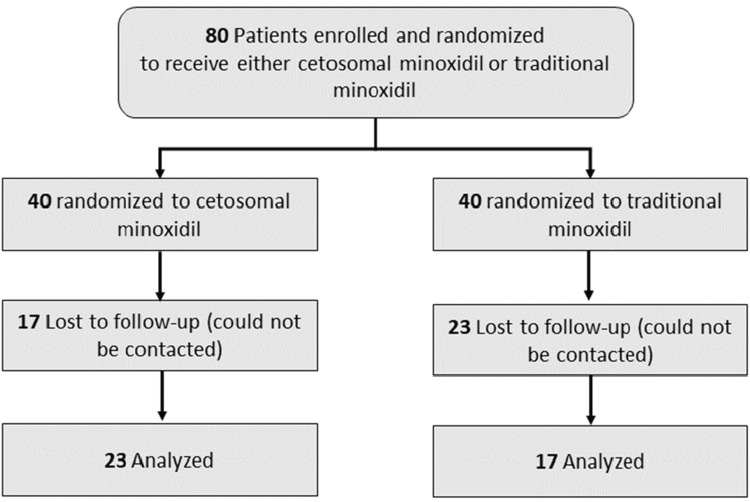
Consolidated Standards of Reporting Trials (CONSORT) flow diagram of the study

Of the 80 patients, 40 were lost to follow-up; the remaining 40 patients completed the study and were considered for a complete analysis (Figure 1).

There was a homogeneous distribution of patients in both groups, as shown in Table 2.

**Table 2:** Baseline demographics SD: standard deviation; AGA: androgenetic alopecia

	5% cetosomal minoxidil	5% traditional minoxidil	p value
N	23	17	
Mean age (SD), years	33.21±9.05	32.46±9.15	0.79
Duration of AGA (SD), years	2.63±1.83	2.60±1.67	0.95
Baseline trichoscopy score	5.92±2.5	6.00±2.44	0.92
Baseline Hairdex-29 score	40.26±4.71	39.64±4.98	0.69

Twelve out of 23 patients (52%) in the 5% cetosomal minoxidil group and four out of 17 patients (24%) in the alcohol-based minoxidil group had a positive increase in hair growth, as determined by the trichoscopy score (p=0.1). In the cetosomal minoxidil group, the baseline mean trichoscopy score was 5.92±2.5, which improved to 7.70±4.07 (30% improvement) at week 16, whereas in the alcohol-based group, it increased to 7.63±4.04 from 6.00±2.44 (27% improvement). This difference was not statistically significant (p=0.95). There was an initial decrease in the mean trichoscopy score in both groups at week 8 (Table 3).

**Table 3:** Assessment of the trichoscopy scores at weeks 8 and 16

Efficacy parameter	5% cetosomal minoxidil	5% traditional minoxidil	p value
Improvement in trichoscopy score, n (%)	12 (52)	4 (24)	0.1
Mean trichoscopy score, 8 weeks	5.36±2.16	5.41±2.11	0.94
Mean trichoscopy score, 16 weeks	7.70±4.07	7.63±4.04	0.95

**Figure 2:**
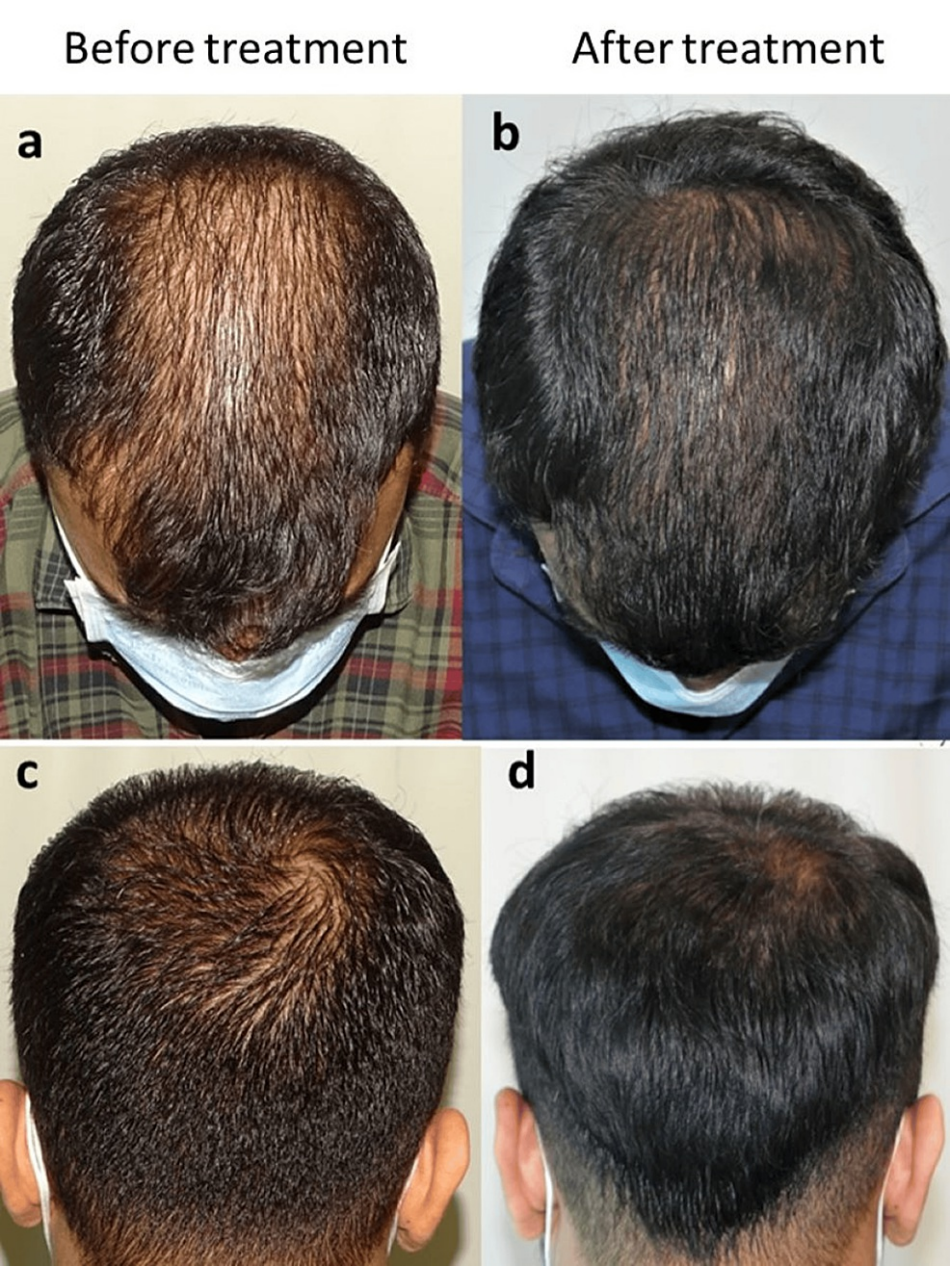
Clinical efficacy of 5% cetosomal minoxidil: a and c, before treatment; b and d, after treatment. Photo courtesy: Dr. Sandeep Sattur

According to the BASP grading system, nine out of 23 (39%) and five out of 17 (29%) patients in the cetosomal and alcohol-based minoxidil groups, respectively, showed improvement (p=0.73). Similarly, 19 out of the 23 patients (83%) and 10 out of the 17 patients (59%) in the 5% cetosomal and alcohol-based minoxidil groups, respectively, reported positive hair growth on the global photography assessment (p=0.15). The clinical efficacy of 5% cetosomal minoxidil is depicted in Figure 2.

All the patients tolerated the treatment well, with no discontinuation in either groups. There were four adverse events (AEs) in the cetosomal minoxidil group, as reported by two (9%) patients, whereas in the alcohol-based minoxidil group, 10 adverse events were reported by seven (41%) patients. Pruritus and flaking were the most common AEs encountered in both groups (Table 4). This difference was statistically significant (p=0.02).

**Table 4:** Adverse events (AEs) in both groups

Adverse events (AEs)	5% cetosomal minoxidil (n=4)	5% traditional minoxidil (n=10)
Pruritus (no. of AEs)	2 (50%)	5 (50%)
Flaking (no. of AEs)	1 (25%)	4 (40%)
Rash (no. of AEs)	1 (25%)	1 (10%)

In addition, as per the Hairdex-29 questionnaire results, hair-related QoL improved in both groups. In the cetosomal minoxidil group, the mean Hairdex-29 score of 40.26±4.71 at baseline improved to 32.32±3.35, whereas it improved to 34.64±3.41 from 39.64±4.98 in the other group. This difference was statistically significant (p=0.03). Overall, 78% and 41% of patients reported improvement in the Hairdex-29 score, respectively (Table 5).

**Table 5:** Hairdex-29 scoring and improvement at week 16

Hairdex-29	5% cetosomal minoxidil	5% traditional minoxidil	p value
Total no. of patients with improvement (%)	18 (78)	7 (41)	0.02
Mean Hairdex-29 score	32.32±3.35	34.64±3.41	0.03

## Discussion

Topical administration of a minoxidil solution is the most common treatment for AGA [11,12]. However, it can be challenging to obtain topical drugs that can enter the scalp. For a drug to be effective, it must (1) primarily reach the scalp and minimize medication loss on the hair or surrounding skin; (2) be readily released from the vehicle; and (3) penetrate the epidermis, outer root sheath of the infundibulum, follicular canal, and layers that surround the hair shaft. Medication must also seem acceptable to ensure compliance, especially if it is administered frequently and over an extended period. Ideally, the formulation should be non-irritating and have a low allergic potential. However, because of the presence of ethanol in the traditional formulation, many commercial solutions can unfortunately induce a wide range of topical skin reactions [7]. To our knowledge, the efficacy and safety of a lipid-based minoxidil solution were not compared with those of an alcohol-based minoxidil solution.

Traditional topical minoxidil formulations have several issues that can result in significant drug loss during application and limited patient compliance, which can ultimately lead to unreliable release control of dosage. Ethanol or propylene glycol was added to regular minoxidil to improve penetration of the active ingredient. These additions lead to an increase in potentially harmful skin side effects that can occur when minoxidil crystallizes after solvent evaporation. Minoxidil crystals cause pruritus, rash, dandruff, and allergic contact dermatitis, which greatly reduce patient comfort and lead to low compliance [7,13]. This prompted the creation of many non-alcohol-based minoxidil formulations that were devoid of these cutaneous side effects [2,8,14,15].

Cetosomes are components of the novel fast-acting transdermal delivery (FADD) method, which is related to the penetration enhancement of pharmaceutical components. The FADD method uses a combination of cetylated esters, cetyl and/or stearyl alcohols, polar solvents, and surfactants to create amphiphilic nanoparticles within a stabilized liquid dispersion. The ingredients in cetosomes improve topical transdermal fluxes of bioactive substances without irreversibly impairing the function of the skin barrier [16]. Cetosomes, one of the most recent drug delivery systems, has been found to improve skin kinetics, boost therapeutic adherence, and lessen the side effects of traditional alcohol-based formulations while also increasing the stability of minoxidil [14].

Many previous studies have investigated the efficacy and safety of 5% minoxidil in the treatment of AGA [4,17-19] and found varied hair growth in 54-62% of patients [17]. However, compared to traditional minoxidil, the unique cetosomal minoxidil seems to have a few advantages, including the absence of propylene glycol (implicit inconvenience) and a quicker time to dry after application. In a recently published real-world analysis, cetosomal minoxidil solutions were found to be effective and tolerable in AGA [20]. In our study, there were no statistically significant differences between the two groups in terms of efficacy for any of the parameters. The mean increase in trichoscopy score at 16 weeks was not statistically significant (p=0.95) between cetosomal and traditional (7.7 vs. 7.63, independently) formulations. In our study, 52% and 24% of patients reported increased hair growth in the cetosomal and traditional minoxidil groups, respectively.

Because of the absence of ethanol in the cetosomal formulation, fewer adverse events were reported. In terms of safety, cetosomal minoxidil was found to be statistically significant than traditional minoxidil, with only four AEs (9%) in the cetosomal minoxidil group compared to 10 AEs (41%) in the traditional group.

Cases with AGA are significantly affected by tone-image satisfaction, with potentially adverse psychosocial factors and a negative impact on their QoL [10,21]. Alopecia has numerous given psychosocial complications, including depression, low tone regard, an altered tone image, and less frequent social engagement [21,22]. Hence, in order to treat alopecia patients effectively, it has been proposed that dermatologists address these psychosocial and QoL difficulties [23]. In our study, we estimated QoL using the Hairdex-29 questionnaire and found that the QoL improved significantly in the cetosomal minoxidil group as compared to the traditional group. In the cetosomal minoxidil group, 78% of patients reported an enhancement in QoL, while the same was reported by 41% in the traditional group, indicating an increase in compliance.

In our small study, we demonstrated that a novel topical cetosomal minoxidil formulation was statistically significant in terms of safety and QoL improvement in patients with AGA, demonstrating efficacy equivalent to that of traditional minoxidil at the same time. A major limitation of this study was the small sample size, with half of patients lost to follow-up. This could be due to the occurrence of the COVID-19 pandemic during the study period. Another limitation was the lack of hair density assessments. Hence, long-term clinical trials with larger sample sizes are required to elucidate the benefits of the newer formulations.

## Conclusions

In this clinical investigation, we compared the efficacy and safety of a novel cetosomal minoxidil formulation with those of a conventional formulation and discovered that the cetosomal formulation had a higher safety profile. As QoL is also significantly affected by AGA, dermatologists must provide patients with appropriate care that addresses not only their hair loss but also the emotional discomfort associated with the disease and its practical effects on their daily lives.
